# Crossroads of consciousness: whose decolonization is it in Nigeria?

**DOI:** 10.3389/fsoc.2025.1535330

**Published:** 2025-05-07

**Authors:** Yusuf D. Olaniyan, Mercy O. Martins

**Affiliations:** Department of Education, University of Bath, Bath, United Kingdom

**Keywords:** decoloniality, autoethnography, hermeneutical injustice, colonial legacies, public sphere

## Abstract

The call for decolonial discourse has increasingly gained global purchase, yet its growing visibility often masks an unresolved question: who possesses the voice and agency to participate in these conversations? This paper tries to answer this question within the context of Nigeria, where the impacts of colonial history persist in education and societal norms. Through an autoethnographic approach, we reflect on our experiences growing up and schooling in Nigeria and, subsequently, the UK for postgraduate education to interrogate how these encounters have shaped our understanding of colonialism and de/coloniality. We propose a novel framework to structure our narratives that maps key decolonial erasure and rediscovery stages. These stages illustrate how systemic barriers within Nigeria’s educational systems obscure colonial histories and hinder decolonial engagement. We appropriate Habermas’s public sphere and Fricker’s concept of hermeneutical injustice as theoretical incisions to illuminate how power dynamics influence the availability of critical spaces for decolonial discussions and how knowledge disparities create interpretive limitations. This study offers insight into the lived dimensions of decolonial engagement, questioning its accessibility and resonance beyond intellectual circles. It also contributes to ongoing efforts to bridge decolonial theory and practice by offering insights for more inclusive educational reforms and public engagement in Nigeria.

## Introduction

The resurgence of decolonization and decoloniality discourses has gained purchase among intellectual circles worldwide (Santos, 2014; Grosfoguel, 2007; Ndlovu-Gatsheni, 2013; Mignolo, 2007, 2009; Fasakin, 2021), foregrounding the urgent need to dismantle colonial legacies imbued within knowledge production and dissemination. Amidst this resurgence, an often ignored critical interrogation is the question: “Who possesses the voice and agency to shape decolonial discourse?” Particularly in post-colonial societies like Nigeria, where the specters of colonialism persistently haunt the corridors of knowledge and power.

While the global rise of decolonial rhetoric has increased calls to dismantle colonial legacies, most of the dialogue is still dominated by intellectual elites and limited by existing power structures. In Nigeria, the question is not only who benefits from decolonial activities, but also who has the voice and authority to engage in these discussions. This study aims to investigate how power dynamics and systemic inequities determine Nigerians’ ability to engage in and influence decolonial discourses.

The study uses an autoethnographic approach to critically explore this question, drawing on the authors’ personal narratives and reflections. It examines how colonial legacies have shaped their lived experiences within educational systems in Nigeria and abroad while unraveling the realities and praxis of decolonial engagement by challenging dominant, monolithic narratives. To structure these reflections, the authors propose a novel framework that captures key stages in the erasure and rediscovery of decolonial consciousness, ringfencing Initial Exposure, Historical Knowledge Vacuum, Curricular Amnesia, Colonial Silencing Mechanism, Identity Disconnection Syndrome and Discursive Erosion. This framework visibilized the systemic paths through which colonial legacies continue to shape educational practices, public discourse, and personal identities in Nigeria.

Growing up in the Western part of Africa, Nigeria, the authors were exposed to an educational system that often overlooked colonial history, discouraged the use of indigenous languages, and prioritized Western epistemologies. These experiences created a sense of cultural estrangement and affected their perceptions of identity, belonging, and knowledge. It was only during their postgraduate studies abroad that they encountered decolonial debates, which sparked a resurgence and greater engagement with coloniality and decolonization issues. The Nigerian context, with its colonial histories and present-day global interdependencies, reflects broader challenges faced by postcolonial societies in the Global South. The exclusion of marginalized voices from decolonial discourse is not unique to Nigeria but exemplifies a global pattern of epistemic inequality that perpetuates colonial hierarchies. Valoma (2024) lend credence to this argument that the hierarchies of domination extend beyond the Global North–South divide, permeating the Global North itself, where minority cultures and languages are often subordinated to dominant ones. An example is the disparities within Global North countries, such as Finland, where the Sámi people’s cultural and linguistic heritage faces marginalization by dominant societal norms (Valoma, 2024) and New Zealand, where the Māori people have experienced marginalization through the suppression of their language and knowledge systems within colonial structures (Smith, 1999).

Unlike existing studies that focus primarily on the academic and theoretical dimensions of decolonization (see Masaka, 2019; Andreotti, 2011; Mbembe, 2016; Ndlovu-Gatsheni, 2018), this paper emphasizes how these discussions permeate—or fail to permeate—the everyday lives and consciousness of the general populace. It challenges the prevailing view of decolonization as a purely academic pursuit by arguing for its integration into everyday life through accessible education, community engagement, and practical reforms. It sets forth the limitations of present discourse and provides pathways for inclusive decolonial practice, with the goal of contributing to Nigeria’s ongoing efforts to reclaim indigenous knowledge, reform curricula, and reimagine societal structures. Without critical intervention, the persistent exclusion of Nigerian voices in decolonial discourse risks reinforcing the colonial hierarchies that these efforts attempt to destroy.

The study uses Habermas’s (1991) public sphere theory and Fricker’s (2007) concept of hermeneutical injustice as theoretical logics to make visible how power dynamics restrict access to critical spaces for decolonial discussions and marginalize interpretive resources necessary for understanding colonial legacies. Habermas’s idea of the public sphere provides a lens to analyze how discourse is shared—or withheld—across different societal groups, while Fricker’s hermeneutical injustice sheds light on the epistemic exclusions that prevent many Nigerians from critically engaging with their histories and identities. Together, these theories reveal how systemic inequalities perpetuate colonial silences and shape public consciousness.

## Decolonization: a quiet discourse or an academic discourse?

Decolonization as a discourse has gained substantial traction in academic institutions around the world. However, we used our autoethnography in this anthology to argue that it remains a rather insulated conversation that often fails to penetrate the broader societal consciousness. Similarly, scholars such as Mbembe (2016), Ndlovu-Gatsheni (2018), and Mignolo (2007) argue that, while decolonization discourse addresses critical questions of knowledge, power, and history, its circulation is frequently limited to the academic elite, raising concerns about its effectiveness in achieving societal transformation. This section critically explores the essence of decolonial discourse as mostly academic, probing the elements that contribute to its restricted reach and weighing the implications for decolonization’s potential as a transformational societal project.

Decolonial discourse originated from intellectual critique of unending postcolonial legacies, with the goal of dismantling power structures which privilege Western epistemologies over Indigenous, African, and other oppressed knowledge systems. Early theorists like Quijano (2000) and Grosfoguel (2007) saw decoloniality as an essential lens for evaluating modernity, which they felt was inextricably linked to colonialism. They argued that colonial structures still shape not just political and economic systems, but also knowledge production and epistemic frameworks (Quijano, 2000; Grosfoguel, 2007). Despite these aims, the conversation on decolonization has typically remained limited to intellectual circles, with professors and academics largely discussing and theorizing rather than addressing grassroots movements or the general public.

One explanation for the academic confinement of decolonial discourse lies in the very institutional frameworks in which it is produced. Santos (2014) and Mbembe (2016) have argued that universities, as colonial-era institutions, still operate within the epistemic confines of coloniality, even as they champion decolonization. Decolonial theorists point to the paradoxical situation in which decolonization is taught, discussed, and developed within universities that replicate the hierarchies and structures that decoloniality strives to demolish (Santos, 2014; Mbembe, 2016; Lorde, 1983). For example, Santos (2014) claims that the university’s role in constructing hegemonic narratives about knowledge makes it fundamentally resistant to the kinds of drastic adjustments that decoloniality requires. This paradox contributes to the academic insularity of decolonial discourse, restricting its resonance to those who have the educational status to access and participate in these theoretical discourses.

Furthermore, the discourse on decolonization is sometimes buried in abstract terminologies and complicated theoretical frameworks, which may alienate non-academic readers. Ndlovu-Gatsheni (2013) stresses that decolonial discourse frequently employs specialized terminology—such as “epistemic disobedience” or “decolonial option”—that assumes knowledge with theoretical concepts that are often unavailable to broader audiences. The thick academic vocabulary associated with decolonial study may create impediments to the discourse reaching populations that are probably the most affected by colonial legacy. According to Ndlovu-Gatsheni (2013), when decoloniality is presented as an abstract academic endeavor, it runs the risk of becoming a “quiet discourse,” one that criticizes without confronting the existing quo in practice.

Furthermore, researchers such as Tuck and Yang (2012) contend that decolonization is routinely co-opted in academic and institutional contexts, where it is depoliticized and shorn of its transformational potential. Tuck and Yang (2012) famously caution against viewing decolonization as a metaphor, arguing that the true goals of decoloniality—land return and the dismantling of colonial systems—are often diluted in academic discourse, rendering decolonization a performative rather than an actionable pursuit. This metaphorical approach can perpetuate decolonization as a primarily intellectual exercise, undermining its intended disruptive force. As Tuck and Yang (2012) argue, when decoloniality is confined to intellectual debate, it risks being subsumed into the very structures it seeks to disrupt, thus remaining “quiet” in terms of practical impact.

The institutionalization of decolonial discourse within academia may also contribute to its limited reach. Bhambra (2014) argues that many universities incorporate decoloniality in superficial ways, such as adding non-Western texts to syllabi or hosting symbolic events that do little to challenge the foundations of knowledge production. This “academic decolonization,” as she calls it, risks becoming a checkbox exercise that satisfies institutional diversity goals without fundamentally disrupting established epistemic hierarchies. Bhambra (2014) warns that such gestures, while well-intentioned, often serve to placate calls for genuine decolonial engagement, thereby maintaining the discourse within the bounds of academic respectability and limiting its transformative scope.

Moreover, the digital era has seen a rise in decolonial discourse across social media and other online platforms, potentially broadening its reach. Yet, as Noble (2018) contends, these platforms come with their own limitations, as algorithms prioritize sensational content over substantive, nuanced discussions. While hashtags like #DecolonizeThis and #RhodesMustFall have raised awareness, these discussions are often reduced to brief, consumable snippets that lack depth, thereby reinforcing decoloniality as a “quiet” discourse even in the digital sphere. The platformed nature of these discussions on social media highlights the tension between the academic and popular realms, with complex decolonial issues often oversimplified to fit digital consumption patterns, thus limiting the critical engagement necessary for societal transformation (Noble, 2018).

The question remains, then, of how to extend decolonial discourse beyond academia and engage with the communities most affected by colonial legacies. Scholars such as Ndlovu-Gatsheni (2018) and Andreotti (2011) advocate for a praxis-oriented approach to decolonization, which would involve translating theoretical insights into actionable strategies within communities. Andreotti (2011) emphasizes the importance of participatory engagement, where communities co-create decolonial frameworks that address their specific needs, rather than passively receiving academic theories. This method reimagines decoloniality as a participatory, collaborative effort, shifting it from a silent, insular debate to one with real societal influence.

Decolonization as an academic discourse raises crucial problems concerning decoloniality’s accessibility and efficacy as a structural change movement. While academia has been a significant place for questioning and theorizing decoloniality, its limitations are becoming increasingly obvious. If decolonial discourse is restricted to academia, it risks becoming a quiet conversation—discussed but ultimately disconnected from the realities of people it tries to empower. To transform decoloniality from an intellectual quest to a lived reality, we must go beyond academia, engage people directly, and challenge the institutional and epistemic institutions that continue to perpetuate colonialism.

## Public engagement with decolonial discourse

The spread of decolonial ideas through public engagement has occurred across various platforms, such as educational institutions, social media, and grassroots activism (Sium et al., 2012; Bhambra, 2014). Universities have emerged as battlegrounds for decolonial initiatives, as seen in efforts to reform curricula and broader calls for institutions to acknowledge and address their colonial histories (Bhambra, 2014). Nevertheless, these efforts face significant obstacles, given that universities, as powerhouses, frequently resist meaningful change (Santos, 2014). Criticisms of the academic decolonization movement often highlight its superficial nature, noting that it sometimes merely involves the addition of a few non-Western texts or symbolic actions rather than a rethinking of the foundational epistemological structures that shape higher education (Mbembe, 2016).

A major challenge in public engagement with decolonial discourse is the risk of co-optation and the integration of its transformative potential. As Maldonado-Torres (2016) cautions, decoloniality could become a trendy ‘buzzword’, devoid of its original intent and superficially adopted by institutions without enacting genuine change. This concern resonates with Tuck and Yang’s (2012) critique, which highlights that “decolonization” is often invoked metaphorically in ways that obscure its true implications. For example, incorporating decolonial language into policy documents or corporate social responsibility agendas can be perceived as an attempt to placate calls for structural reform without addressing the fundamental issues of coloniality.

Additionally, public engagement with decolonial discourse frequently faces substantial pushback. Initiatives like Rhodes Must Fall, and efforts to dismantle colonial-era monuments have ignited intense debates, with opponents contending that such actions amount to erasing history and cultural heritage. These disputes expose the strong emotional and cultural ties to colonial symbols and the difficulties in reaching a shared understanding of what decolonization means in practical terms (Pillay and Swanepoel, 2018). The polarized reactions to these movements show the complexities of involving the public in discussions about decoloniality, as there is often a marked reluctance to acknowledge the uncomfortable truths about the persistent impact of colonialism. Ngaruiya et al. (2024) argue that the Global South must actively anchor the change in decolonial conversations rather than passively awaiting decisions from the Global North.

Digital platforms have emerged as critical sites for public involvement with decolonial discourse, providing both advantages and disadvantages. Social media has made it easier to spread decolonial ideas around the world and build virtual communities that cross borders (Ndlovu-Gatsheni, 2018). Hashtags like #DecolonizeThis, #RhodesMustFall, #WhyismyCurriculumWhite and #EndSARS in Nigeria described by Strong and Nafziger (2021) as “a part of a wider movement for black lives, African emancipation from neo-colonialism and globalized antiblack racism, for global unity, and the reignition of class struggle.” (p. 45) have helped activists and researchers connect and collaborate, amplifying underrepresented perspectives and promoting a global conversation on decolonial issues (Bosch, 2022). However, the digital environment also poses obstacles. Algorithms that promote sensational content, the dissemination of misinformation, and the echo chamber effect can all impede nuanced debates of decolonial theories (Noble, 2018). Furthermore, the digital divide implies that access to these online places is uneven, frequently excluding individuals from the Global South who are most affected by colonial legacies (Gajjala et al., 2022; Gajjala, 2019). While digital platforms have increased access to decolonial discourse, they could also reproduce or strengthen existing inequities because of their agentic potential of being digital colonizers.

Furthermore, art and cultural production have been instrumental in involving the public in decolonial discourse. Forms of artistic expression such as literature, visual arts, and performance have served as powerful means of challenging colonial narratives and imagining alternative futures (Mirzoeff, 2017). Events like the 2022 Documenta 15, an art installation-based exhibition, which focused on decolonial themes, have brought these conversations to a wider audience, encouraging critical examination of the colonial histories embedded within cultural institutions themselves (Lettau and Canyürek, 2024; De Oliveira, 2023). Documenta 15, curated by the Indonesian collective called ruangrupa, centered on the concept of ‘lumbung’ (communal sharing), that challenges Western-centric curation. The installations by the Wajukuu Art Project in Kenya and Sa Sa Art Projects in Cambodia, highlighted anti-colonial resistance and alternative epistemologies (Lettau and Canyürek, 2024). However, the commercialization of decolonial art creates a paradox. As artists attempt to engage the public with decolonial concepts, their work often gets absorbed into the capitalist market, which can dilute its oppositional message (McEvilley, 1999; Thomas, 2022). This situation mirrors the challenge of preserving the authenticity of decolonial engagement within a neoliberal system that tends to prioritize profit over meaningful social change (Thomas, 2022).

Musicians in Nigeria, such as Fela Kuti and Burna Boy, have utilized their platforms to promote decolonial discourse in an accessible and non-hierarchical ways. Fela Kuti, as argued by Saleh-Hanna (2008), used his music to critique colonial legacies and challenge the behaviors of African elites and broader Nigerian society. His songs condemned the tendency of Africans to emulate colonial cultural practices, such as adopting Western dress styles, even after achieving independence. This critique aligns with Albert Memmi’s seminal work ‘The Colonizer and the Colonized’ (Memmi, 1957), which examines the persistent psychological and cultural impact of colonization. Beyond his music, Fela also embodied decolonial values through his distinctive Afrocentric attire and speech, reinforcing his rejection of colonial norms.

Similarly, Burna Boy addressed the enduring effects of colonialism through his song ‘Monsters You Made,’ released in October 2022. Osibodu (2023) noted that Burna Boy critiques the British-influenced Nigerian education system, particularly its lack of engagement with critical historical truths. His lyrics, such as “the teacher dem teaching what the white man dem teaching” (Osibodu, 2023, p. 182), highlight the continuation of Eurocentric curricula in Nigerian schools. Burna Boy efficiently engages multiple audiences and emphasizes his work’s decolonial message through translanguaging, which involves switching between English and Nigerian Pidgin.

Authors such as Chinua Achebe and Wole Soyinka have used different literary forms to explore postcolonial reality. Achebe uses his novels like Things Fall Apart (Achebe, 1958) and No Longer at Ease (Achebe, 1963), to critically examine the sociocultural disturbances generated by colonialism and its aftermath. In contrast, Wole Soyinka, a dramitist, frequently depicts the complexities of the Nigerian (and Western-influenced) culture through a different narrative method to explore these subjects. His plays, such as A Dance of the Forest (Soyinka, 1964), confronts the realities of colonial experience and how it has shaped post colonial settings through intense dialogue Together, these authors use their respective mediums to confront colonial legacies and foster a collective consciousness about cultural reclamation and resistance. While Achebe’s work conscientize and provoke thinking and reflections, Soyinka’s theatre creates immediate engagement. Similarly, Boal (1979), in his work Theatre of the Oppressed, particularly through Forum Theatre, creates a space similar to Habermas’ public sphere, where people can engage with their lived realities. His work, applied in some educational spaces, serves as a means of creating decolonial conversations that cut across sectors. Dramatists like Osofisan (1982) and Sowande (1979), with works like More the Wasted Breed and Farewell to Babylon, also shed light on the injustices of colonialism and call for resistance through drama.

Similarly, community engagement, especially through decolonial pedagogy, has been a vital part of promoting public involvement with decolonial discourse. Grassroots initiatives, workshops, and public lectures have been utilized to educate communities about the historical and ongoing effects of colonialism and to explore practical strategies for decolonial action (Andreotti et al., 2011). These efforts typically embrace a praxis-oriented approach, focusing on collective learning and action to disrupt colonial power structures (Smith, 1999). However, they encounter several challenges, such as limited resources, institutional resistance, and unstable funding for community-based projects (Tuck and McKenzie, 2015). Moreover, the success of decolonial pedagogy often depends on participants’ willingness to confront uncomfortable truths about their own roles in sustaining colonial structures (Grande, 2018), which can be particularly difficult in environments where nationalist or settler-colonial identities are deeply rooted.

Ultimately, public engagement with decolonial discourse is a difficult and contentious issue. Although there has been some success in expanding awareness of decolonial ideas, significant challenges such as co-optation, resistance, and institutional disparities remain. The future of decolonial engagement is dependent on keeping its critical edge and serving as a true force for transformation, rather than becoming a diluted buzzword. This will necessitate ongoing efforts to hold institutions responsible, promote open and accessible dialogue spaces, and support grassroots groups spearheading decolonial endeavors. As decolonial thought evolves, scholars, activists, and communities must be cautious against co-optation and struggle for actual transformation in both theory and practice. For example, how can students in primary and secondary school actively practice or embody decoloniality or be anti-colonial in spaces other than the classroom when they are punished or fined for speaking anything other than colonial English in the school environment? How can they have access to what tertiary academics or decolonial scholarship have to offer when the library is filled with colonial English text? The present reality is still the reality of the authors over a decade ago, with many changes in policies yet, no practical enforcement. This actions or inaction continues to elevate colonial ideologies and in general its legacies while marginalizing and suppressing local thoughts and ideologies in the school. This is particularly important as school reproduces societal norms in what we term as an unending coloniality cyclic loop.

## Theoretical engagements: Habermas public sphere and Fricker hermeneutical injustice

In this research, we draw on Habermas’s theory of the public sphere and Fricker’s concept of epistemic injustice to explicate the dynamics of decolonial discourse in Nigeria, particularly how unequal power relations shape the understanding and dissemination of this discourse among different societal groups.

Habermas (1991) concept of the public sphere refers to a space where people can gather to discuss and debate issues of common concern, ideally free of governmental and market pressures. In this context, rational-critical discourse is expected to create public opinion, which can then affect political action. In this research, the concept of the public sphere provides a useful framework for analyzing how decolonial discourse is—or is not—articulated and shared among different societal groups. Certain groups, such as academics, activists, and intellectuals, who have the educational and social capital necessary to engage with these complex ideas are primarily responsible for driving the decolonial discourse. However, these discussions often do not reach the broader populace, who may lack the resources or platforms to participate meaningfully in these debates. This uneven participation reflects a fragmented public sphere, where some voices are amplified while others are marginalized, thus creating a polarized discursive field that challenges the democratic ideal envisioned by Habermas.

In this research, we used Habermas’s theory to highlight the importance of creating more inclusive spaces for decolonial dialogue and its translative impact in Nigeria. We argue that for the public sphere to effectively create an inclusive public opinion on decolonization, it must be accessible to all societal groups, especially those who have been historically marginalized or excluded from intellectual and political conversations. Achieving this requires expanding the reach of decolonial discourse and addressing structural obstacles like inadequate education, limited media access, and language barriers, which restrict broader participation. Miranda Fricker’s idea of epistemic injustice, particularly her concept of hermeneutical injustice, provides an additional viewpoint on the dynamics of decolonial discourse in Nigeria. Fricker (2007) distinguishes between two sorts of epistemic injustice: testimonial and hermeneutical. Testimonial injustice occurs when bias unjustly diminishes a speaker’s credibility, whereas hermeneutical injustice emerges from a lack of communal interpretative resources, resulting in misunderstanding or marginalization of particular social experiences and groups.

We used hermeneutical injustice in this work to address the unequal distribution of interpretive resources required to understand and explain one’s social experiences. Certain groups, i.e., academia, researchers, and NGOs in Nigeria have established a discourse on coloniality and decolonization, and they have the intellectual tools and historical knowledge to engage with these themes. However, for many others, particularly those without access to higher education or who are outside of academic and activist groups, these discussions may appear distant or unconnected to their personal experiences. Because of this interpretive resource deficit, their viewpoints and experiences with coloniality may go unspoken or misinterpreted in the larger debate. Fricker’s approach emphasizes the power dynamics involved in the spread of decolonial knowledge in Nigeria. It implies that people who dominate the discourse are not just the most outspoken, but also have the interpretive frameworks to understand and transmit these complicated ideas. This monopolization of interpretive resources leads to epistemic exclusion, in which the experiences and understandings of less privileged groups are rendered invisible or invalid.

## Methodology

In this study, we use an autoethnographic approach, a qualitative research method that combines autobiography with ethnographic analysis (Chang, 2016). Ellis (2004) describes autoethnography as “research, writing, story, and method that connect the autobiographical and personal to the cultural, social, and political” (p. 19). This methodology enables us to investigate how decolonial discourses permeate—or fail to permeate—beyond the academic circles, as well as how they are accepted and engaged with by the general public in Nigeria. We critically examine whether these discourses have been effectively mobilized to reach a broader audience or if they remain limited to intellectual and scholarly debates with minimal societal impact. We leverage our own experiences as a lens for analysis and seek to identify the key players (communities, parents, teachers, friends, schools, religious houses, hospitals…) involved in promoting, resisting, or engaging with decolonial initiatives and to assess the role of various societal actors in advocating for or challenging these projects.

Our exploration began with a reflective review of our lives as people born and raised in Nigeria as well as our educational journey both in Nigeria and overseas. The two authors have distinct identities in Nigeria, molded by their upbringing and experiences in different geopolitical zones. They belong to different regions with varying languages, religions, cultures and ethnicities. We have lived, studied, and worked in practically all six of Nigeria’s geopolitical zones. This diverse exposure informs our reflections and allows us to capture the rich complexities and nuances that characterize these regions. Our perspectives, therefore, provide a good representation of Nigerian society, showcasing its cultural and regional diversity. Both of us finished our primary, secondary and undergraduate studies in Nigeria, and it was not until we pursued postgraduate studies in the United Kingdom that we became aware of decolonial discourse and its conversations. One of us worked as a primary school teacher, and the other worked as a teacher, later interning with a multinational organization. This personal interaction with decolonial conversations prompted us to reflect on our experiences as students and teachers in the Nigerian educational system, and we documented our thoughts and observations using digital journaling and highlighter tools, which allowed us to capture real-time reflections and highlight significant themes as they emerged (Makaiau et al., 2018). These tools provided a flexible and accessible platform to engage in a continuous process of reflection and analysis, while also allowing us to revisit and refine our insight overtime especially during conversations. This reflection took place over 3 months, during which we used a shared Microsoft document as an online journaling platform independently at first and met on a bi-monthly basis to harmonize our ideas, noting similarities and differences.

Autoethnography is especially suited to our research, as it enables us to explore the subjective experiences of navigating systems shaped by colonial legacies and global modernity (Ellis, 2004). With the examination of our own narratives, we aim to uncover the complexities of engaging with these structures and offer perspectives that are often absent in traditional research methods (Chang, 2016). The digital highlighter tool, in particular, added an interactive dimension to our reflection process, helping us identify recurring themes across our personal narratives. However, this approach also presented certain challenges, as the digital tools sometimes created a sense of detachment from the emotions captured in our reflections.

Given our dual roles as researchers and participants, reflexivity was vital to our study process. Throughout the project, we engaged in continuous self-reflection, challenging our beliefs and examining how our positionalities influenced the information we produced. As people who had been through both the Nigerian educational system and the Western academic context, we were aware of how our backgrounds could influence our ideas on decoloniality, and the use of digital highlighters also served as a reflexive tool, prompting us to consider why we chose to emphasize certain aspects of our experiences and what this revealed about our own biases. Despite these reflexive practices, we frequently confronted doubts about the objectivity and reliability of our findings. Autoethnography inherently involves subjective interpretation, and we grappled with the emotional and personal nature of our analysis. This was particularly challenging when reflecting on moments that underscored the enduring colonial influences within our educational experiences. The digital highlighter allowed us to isolate these moments, but it also raised concerns about how selective highlighting could shape our analytical focus.

To mitigate these challenges, we adopted a strategy of critical distancing, which involved revisiting our journal entries and digital highlights regularly to separate our initial emotional responses from the analytical insights we were seeking to develop. This process helped us to situate our personal experiences within a larger theoretical framework of decoloniality, linking individual narratives to broader systemic issues. At times, we found that our emotional engagement was essential to understanding the impact of coloniality on our experiences, and we actively worked to balance this emotional involvement with analytical rigor, recognizing that both are crucial for a comprehensive understanding of decolonial processes.

Throughout this research, we remained mindful of ethical considerations, particularly given the personal and often sensitive nature of our reflections. We were careful to protect the identities and confidentiality of individuals indirectly referenced in our narratives, ensuring that our self-disclosures did not compromise others. The emotional challenges of this research were significant; reflecting on experiences that revealed colonial legacies in our education often elicited feelings of frustration, anger, and disillusionment. These emotions were particularly intense when revisiting highlighted entries that brought deeply personal insights to the surface. To manage this, we employed strategies to balance emotional engagement with analytical rigor. One approach was to regularly review our highlighted content, making a conscious effort to distinguish between emotional reactions and the insights we were aiming to develop. This practice allowed us to reframe our experiences within the broader context of decolonial theory and identify connections between our personal narratives and systemic issues.

Moreover, we maintained a routine of continuous self-reflection, where we questioned our assumptions and acknowledged how our dual roles as researchers and participants shaped our perspectives. By embracing both subjectivity and objectivity, we aimed to navigate the complexities inherent in decolonial research, recognizing that both are essential components of the process. For our data analysis, we used thematic analysis to systematically interpret our journal entries and digital highlights. This method enabled us to identify recurring themes related to colonial legacies and their influence on educational practices, thus allowing us to connect our personal experiences with broader theoretical discussions (Braun and Clarke, 2022). We decided to use a model to guide our autoethnographic narrative to ensure that our experiences were constantly triangulated with decolonial theory, praxis and our experience while ensuring that the often-sidelined realities fuelled by the systemic failure and/or coloniality were visibilized in the narratives. While the digital highlighter facilitated the efficient organization and grouping of themes, it also occasionally created a sense of distance from the rawness of our experiences. To counter this, we made a point of revisiting both highlighted and unhighlighted sections to ensure that our analysis remained grounded in the entirety of our narratives.

This methodological approach, incorporating digital tools such as journaling and highlighting, enabled us to engage deeply with our experiences while situating them within the broader decolonial framework. We found that this balance between personal engagement and critical analysis was essential for advancing the goals of decolonial research and contributing to social justice.

## Proposed autoethnography model

**Figure:**
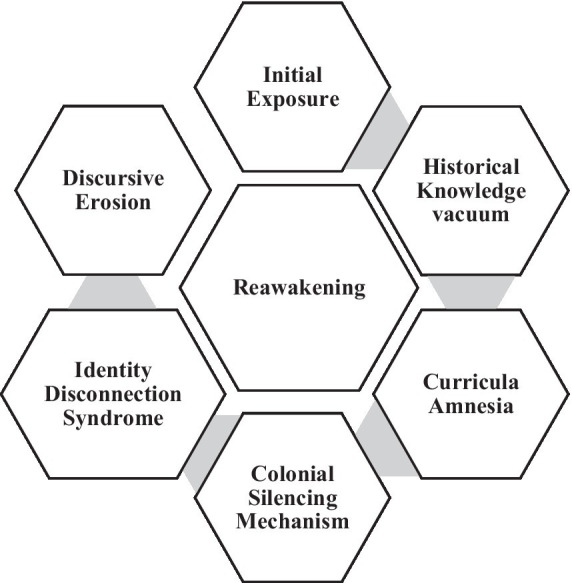


### Autoethnography

In this critical autoethnography, we reflect on our journey through the Nigerian educational system, drawing on personal experiences to uncover how decolonial conversations have been systematically excluded, often dismissed or trivialized as irrelevant due to the perception that colonialism is a thing of the past, thereby erasing awareness of its enduring legacies. Nigeria currently operates the 9–3–4 education system (9 years of basic education, 3 years of senior secondary education and 4 years of tertiary education). However, we were schooled when the 6–3–3-4 system was in practice (6-year primary education, 3-year junior secondary, 3-year senior secondary and 4-year tertiary) (Federal Government of Nigeria, 2013). While these change in structural models were introduced as reforms to expand access and transform the education sector to conform with international initiatives like the MDGs, which are now the SDGs, and the Education For All goals (Ukpong et al., 2023), our reflections suggest that these frameworks did little to challenge the epistemological foundations inherited from colonial rule. Ajibade (2019) also critiques the transformation of 6–3–3–4 to 9–3–4 systems, highlighting the emphasis on structural change and certification over what Mignolo (2009) called “epistemic decolonialization.”

Our educational journeys, shaped by these structural reforms and their inherited colonial underpinnings, thus serve as entry points for interrogating how curriculum content, language policy, and institutional culture at each level of schooling reinforced or obscured decolonial thought.

Throughout our educational experiences, from primary, secondary and undergraduate studies in Nigeria to postgraduate pursuits abroad, we became increasingly aware of how key historical and decolonial narratives were absent in our formative years, leaving us disconnected from a significant part of our cultural and historical identity. By drawing on existing theories and integrating them with each stage of our experience, we aim to explore how decolonial discourse has been obscured from mainstream education and how we have come to re-engage with these conversations in a new academic context.

This discussion is informed by a model that we created to map out our journey, identifying stages that highlight the systemic barriers to engaging with decolonial ideas. Each stage reveals distinct mechanisms by which our educational experiences limited our understanding of coloniality, from Initial Exposure to Discursive Erosion and Reawakening. We adopt an autoethnographic approach to analyze these stages in relation to the broader academic discourses on decoloniality and epistemic injustice, as discussed by scholars such as Mbembe (2016), Fricker (2007), and Ndlovu-Gatsheni (2018). Our methodology is inspired by Ellis’s (2004) definition of autoethnography as a way to connect the autobiographical with cultural, social, and political narratives, allowing us to critique the structural erasures we have encountered.

Our early educational experiences in Nigeria provided little more than a baseline exposure to history, mainly through informal sources like family stories and community discussions. Formal schooling, however, did not begin to address the complexities of coloniality or offer a critical perspective on Nigeria’s colonial past. This stage, which we label *Initial Exposure,* represents a time of moderate awareness, grounded more in the personal narratives we encountered outside the classroom than institutionalized knowledge. This initial stage of exposure, or rather lack of critical exposure, reflects a fractured ‘public sphere’ as Habermas (1991) theorizes, where access to discussions about colonial histories is limited to distant select groups. According to Habermas, a healthy public sphere should allow all members of society to engage in rational discourse on matters of shared concern. Yet, by omitting colonial histories, the educational curriculum fails to support a public sphere in which decolonial discourse is accessible, thus restricting our early understanding. Furthermore, this aligns with Fricker’s concept of hermeneutical injustice, as the lack of critical colonial narratives in education leaves students without the interpretive resources necessary to understand or critique colonial legacies from an early age. We both remembered how, from primary school, we were instructed and, in some cases, punished because of the English language. As children, we were made to understand that English was the only language acceptable in the school and a language for success. We can vividly recollect that we were not informed or made to understand why the English language was more important than our local language; instead, these practices created an internalized inferiorization of our local language, a reality that contributed to a series of identity crisis due to the inability of one of the authors to speak their mother tongue.

At this stage, any awareness we had of colonial history was rudimentary and filtered through narratives that often lacked critical engagement. An example we collectively drew on was the third placing of our local names. While we represent the diverse ethnolinguistic groupings in Nigeria, reflected through our indigenous names, most of us were made to answer our English/Arabic names in school and subsequently as first names, with our local names relegated to the position of other names. At some point, we became more comfortable to be referred to by names that aligned with global ‘buzz’ and distanced ourselves and our identity from our local names that bore deep indigenous meaning. This is even reflected in this article as one of the authors retained his Arabic name as his first name and then his local name as the second. The second author, however, only retains her English name as her first name, with her local name represented with just a letter initial. In school, history classes, while present, glossed over colonial impacts, seldom addressing the enduring legacies of colonial power structures in contemporary Nigerian society. Instead, there was an emphasis on the glorification of post-independence nationalism, which subtly marginalized the more painful aspects of colonial exploitation (Ndlovu-Gatsheni, 2018). This aligns with the concept of Curricular Amnesia, wherein colonial narratives are deliberately minimized or omitted, as theorized by scholars who discuss how colonial histories are selectively forgotten or erased in educational contexts (Andreotti, 2011).

In our primary school years, the historical content we were taught was superficial, often celebrating independence without addressing the rudiments of independence, but we were never taught the full scope of colonialism. We knew that dates like Independence Day were holidays because they marked important events, but we did not critically engage with what Nigeria was truly being freed from beyond political freedom. The curriculum focused on memorizing dates and slogans, rather than encouraging us to question the underlying structures of power. As we progressed through our education, the *Historical Knowledge Vacuum* became increasingly apparent. This stage captures the moment when we realized that our curriculum omitted significant aspects of our history. Formal history classes in Nigeria rarely discussed colonial exploitation or its ongoing impacts, creating a vacuum where such knowledge should have been imparted. According to Masaka (2019), this omission is a strategic form of silence that postcolonial states sometimes adopt to avoid grappling with painful legacies that could challenge nationalist narratives. In omitting key historical narratives, the curriculum contributes to what Habermas might call a restricted public sphere, one that prevents students from engaging in full discourse on matters of historical consequence. By excluding discussions on colonial impacts, the system limits access to interpretive frameworks, perpetuating what Fricker describes as hermeneutical injustice—a situation in which individuals are prevented from understanding their social realities due to a lack of interpretive resources. This exclusion not only contributes to Curricular Amnesia but also to a broader cognitive detachment from our own histories. An example of this erasure is the glossing over or tokenistic approach to teaching the historical antecedents of the Nigerian Civil War (1967–1970). First, only students pursuing politically oriented courses had the chance to discuss this topic in class, often framed as a “past is past” narrative disconnected from the present. During our time, subjects like Government did not adequately explain the causes of the Nigerian Civil War, particularly in relation to contemporary secessionist movements such as the Biafra agitation. A recent parallel is the agitation by the Yoruba secessionist group, known as the ‘Oduduwa Republic,’ which challenges the amalgamation of diverse regions into the entity called Nigeria by British colonizers (Akano, 2024).

Just as the reasons for the Civil War and the Biafra and Oduduwa movements are sidelined, the quiet whispers and deep-seated desire to “go our separate ways” remain misunderstood by much of the public. This lack of understanding arises because these critical conversations are not afforded space in educational settings or Habermas’s concept of the public sphere. This exclusion limits the interpretive framework of both educated and uneducated Nigerians, effectively reinforcing Fricker’s notion of hermeneutic injustice.

On the other hand, such conversations occasionally surface on social media, where various agitating groups articulate their grievances and debate their positions. However, we argue that discussions of this magnitude, centered on the nation’s history, when relegated to the sidelines, lead to a public that, due to limited knowledge and the failings of both educational institutions and other platforms, views these movements as radical or merely political lobbying efforts. This prevents critical thought and meaningful engagement with these issues. While we do not aim to justify these movements, we contend that it is an injustice for Nigerians to remain oblivious to the colonial legacy that underpins these grievances.

In this vacuum, the narrative that emerged was one of *Curricular Amnesia*. We recall history classes that presented a sanitized version of Nigerian history, largely focusing on pre-colonial achievements and post-independence politics while omitting discussions of colonial brutality and its ongoing effects. This exclusion reflects what Masaka (2019) describes as an attempt to reinforce a simplified and sanitized national identity, avoiding topics that could lead to dissent or raise uncomfortable questions about postcolonial governance. This aligns with Andreotti’s (2011) argument that education systems shaped by colonial histories often avoid decolonial topics, fearing they might destabilize existing power structures or provoke critical engagement.

At the secondary level, this silence became more institutionalized. History as a subject was optional and poorly resourced. One of the authors recalls that, even when history was taught, it focused on pre-colonial kingdoms like the Alaafin of Oyo, The Benin Kingdom and the Sultan of Sokoto and post-independence nationalism with little or no mention of colonial atrocities or their enduring effects. Instead, subjects like Government and Social Studies pulsated a “unity in diversity” narrative that glossed over the fractures left by colonization. This absence, we argue, is not accidental but a deliberate structuring of ignorance. Similarly, during our undergraduate education in Nigerian universities, the disciplinary frameworks we encountered continued to exclusively center Western paradigms. One of the authors recalls that in his psychology and philosophy of education classes, particularly when discussing child development and classroom management, the theories presented were predominantly Western. The translative effect of this resulted in the thinking about the epistemic legitimacy of African intellectuals to theorize concepts through the lens of African experiences and realities? Moreover, the author who was in the department of Political Science and International Studies recalled that the foundational theories and scholars were almost exclusively European. Even topics on African development were often analyzed through dependency theory or modernisation frameworks, with minimal reference to decolonial thinkers or indigenous perspectives. We were rarely, if ever, encouraged to question the colonial foundations of these epistemes. In retrospect, this amounts to what Mbembe (2016) describes as *intellectual desertification*—a systematic stripping away of tools that could help us critique the coloniality embedded in the very knowledge we consumed.

The next stage in our educational journey involved a recognition of the *Colonial Silencing Mechanism*, a deliberate strategy by which our curriculum continued to exclude any serious engagement with decolonial ideas or critiques of colonial structures. As we advanced to higher levels of education, we encountered an education system that was systematically designed to maintain colonial narratives by silencing alternative perspectives. This sustained silencing of decolonial perspectives deepened what we have termed ‘*Identity Disconnection Syndrome*,’ a sense of detachment from our cultural and historical identity. Fricker’s concept of hermeneutical injustice provides insight here, as the absence of interpretive tools effectively alienates students from their own identities by preventing a full understanding of their historical context. Furthermore, Habermas’s public sphere mirrors the impact of this exclusion, as our education lacked the open, discursive space necessary for meaningful engagement with diverse historical narratives. The result is a public sphere within the educational system that reinforces dominant narratives and marginalizes critical, decolonial discourse.

One of the authors reflects on how the Colonial Silencing Mechanism and Identity Disconnection Syndrome continue to impact them. As a student in the humanities and social sciences, her first encounter with decolonial ideas and the concept of coloniality only occurred during her postgraduate studies abroad. Despite years of undergraduate education in Nigeria, these conversations were entirely absent from their academic experience. This contrast became particularly evident during her time in the UK, where she met fellow international students from diverse backgrounds. Among them were researchers from East Africa who frequently communicated with each other in Swahili, a shared language that transcended national boundaries and created a sense of cultural connection. By comparison, her own Nigerian identity felt fractured. Growing up, speaking local languages in school was discouraged and sometimes punished, leaving her unable to communicate fluently in her mother tongue. This disconnection was magnified when East African peers casually remarked on her inability to speak any Nigerian language, questioning what distinguished her as Nigerian beyond her appearance. This experience of cultural alienation extended beyond language. To assert her Nigerian identity, she felt compelled to adopt stereotypical markers, such as wearing traditional attire, but even this felt inadequate. It ringfenced the deep impact of colonial legacies, which stripped her of the ability to fully engage with her heritage through the various colonial silencing mechanisms embedded in her education and surroundings. The suturing intersectionality of her cultural identity disconnection and belonging informed her research engagement on decolonization. This brings to the fore how coloniality continues to shape personal and academic experiences. We reflected on her experiences and realized that this struggle is not an isolated issue; many individuals grapple with their identity after passing through the colonial silencing mechanisms embedded in Nigeria’s education system.

By the time we reached the latter stages of our undergraduate education, we had fully entered the phase of *Discursive Erosion*, where the prolonged absence of critical perspectives on coloniality began to erode our ability to engage in such discussions altogether. This erosion reflects what Mbembe (2016) refers to as the intellectual desertification of postcolonial thought—a state in which the critical tools needed to analyze and challenge colonial legacies are systematically stripped away. As our educational journey progressed, the consistent exclusion of decolonial narratives led to what we term ‘Discursive Erosion’ and eventual ‘Cognitive Colonization,’ wherein our thoughts were increasingly shaped by unexamined colonial narratives. Habermas’s public sphere emphasizes the role of diverse discourse in shaping an informed public; here, the absence of critical tools meant that no such diversity of thought was accessible, leaving us with a singular, colonial viewpoint. This aligns with Fricker’s notion of epistemic exclusion: by limiting decolonial perspectives, the curriculum systematically marginalized certain ways of knowing, leading us to internalize a view of history that excluded our own cultural context. The culmination of our educational experiences in Nigeria can be described as *Epistemic Shadowing*, a state in which the lack of access to decolonial perspectives cast a shadow over our understanding of coloniality. This stage represents the lowest point in our awareness, as the absence of decolonial narratives left us unaware of the full scope of colonial impacts. In this shadowed state, it was difficult even to perceive the missing perspectives, as our cognitive frameworks had been shaped by years of omission and erasure. Our exposure to decolonial discourse during postgraduate studies abroad marked a significant ‘*Reawakening*,’ as we were finally able to engage in an inclusive academic environment. This aligns with Habermas’s concept of a functional public sphere that supports diverse voices and enables transformative critical discourse. In this environment, we could finally access the interpretive tools to understand and critique colonial legacies—a shift that Fricker would describe as an achievement of epistemic justice. By reclaiming these interpretive resources, we could now reframe our identities and educational experiences within a context that had previously been inaccessible.

This reawakening reshaped our research focus and engagement, and helped us to recognize the connections between identity, belonging, and academic interests. Our work now emphasizes raising awareness of often-ignored aspects of education shaped by colonial histories. We aim to ensure that voices and stories from marginalized communities are included in the conversation. Currently, we are researching Nigeria, specifically secondary and university education. The first author is working with three universities to understand how colonialism and coloniality influence the spatiality of Nigerian Universities and student access. Meanwhile, the second author is researching how Nigerian secondary schools are using English language policy to enact punitive measures on the students. Her goal is to help them reflect on how these policies often prioritize colonial languages while devaluing local ones and encourage them to resist such biases by creating awareness.

Our research strives to create spaces for open and inclusive conversations, which is what Habermas calls a “public sphere.” These are places where people can come together to exchange ideas and challenge existing systems. We focus on building these spaces in schools and universities and share our findings on platforms that are accessible to a wider audience. By bridging these worlds, we hope to bring hidden stories to light and encourage changes that make education more inclusive and fairer for everyone. Our goal is to break down the barriers that continue to marginalize certain voices, creating an educational system that values and respects all perspectives.

Our journey from Initial Exposure through Reawakening highlights the systemic ways in which colonial narratives continue to shape educational experiences in Nigeria and reinforce the need for broader decolonial engagement beyond the academic circle. With our reflections on our journey, we see the need for an educational system in Nigeria that embodies the inclusivity of Habermas’s public sphere—one where decolonial discourse is freely accessible to all. Such a system would support Fricker’s concept of epistemic justice, ensuring that interpretive resources are available to understand Nigeria’s colonial past and its ongoing impacts. Achieving this will require reforming curricula to engage critically with colonial histories, thus empowering future generations to participate meaningfully in decolonial conversations.

## Conclusion and recommendations

This research highlights the critical need for a systemic shift in how decoloniality is understood and enacted in Nigeria. Despite an awareness of this struggle, much of the discourse remains confined to intellectual and elite circles. The failure of decolonial concepts to permeate the general population reflects the entrenched power structures that decoloniality seeks to dismantle. As Fricker’s concept of epistemic injustice demonstrates, these structures systematically remove the majority’s ability to engage critically with their histories, leaving them vulnerable to selective colonial narratives.

To break this cycle, it is essential to move beyond theoretical discourse into praxis-driven initiatives. First, the educational system must be restructured to integrate decolonial curricula at all levels. This requires developing teaching materials that prioritize African histories, indigenous knowledge, and local contexts while deconstructing colonial narratives. Such curricula should be co-created with educators, historians, and community leaders to ensure their relevance to Nigerian realities.

Public forums and community dialogues are also crucial to creating inclusive spaces for critical engagement with decolonial issues. These could take the form of town hall meetings, workshops, and cultural festivals that push decolonial discourse beyond academic institutions into popular consciousness. Schools, through clubs and societies, can also foster spaces for discussing practices that sustain colonial legacies. Habermas’s concept of the public sphere does not necessarily have to operate outside institutions; such spaces can be cultivated within them to encourage critical dialogue on shared concerns.

Digital platforms should be leveraged to broaden access to decolonial ideas and challenge dominant colonial narratives. While digital media may oversimplify complex issues, it offers a powerful tool for connecting movements, sharing indigenous perspectives, and building solidarity. Social media, podcasts, and online resources can make decolonial knowledge more accessible, especially to younger generations.

Revitalizing indigenous languages and knowledge systems is equally essential. Language shapes identity and thought; therefore, policies supporting indigenous language education, oral history preservation, and cultural expression are foundational to decolonization. Additionally, cultural and educational changes must be accompanied by practical economic and policy reforms to address systemic inequities rooted in colonial legacies. Land reform, resource allocation, and economic policies that prioritize historically marginalized communities are necessary for a holistic decolonial praxis. Decolonization must lead to material changes in the lives of those most affected by colonial exploitation.

The task ahead is immense and multi-dimensional. Decolonization cannot remain a theoretical discussion among academics; it must become a lived reality, integrated into education, cultural practices, and public discourse. Reclaiming indigenous knowledge, re-centering African histories, and restructuring curricula are necessary but insufficient without a broader societal reawakening—a shift in how Nigerians see themselves and their place in the world. This is about more than revisiting the past; it is about creating a future that is intellectually autonomous, culturally grounded, and politically free of colonial domination.

The Nigerian decolonial project must transcend academia and evolve into a populist movement—a collective consciousness that confronts colonial epistemic structures. It must involve unlearning historical falsehoods, regaining agency, re-legitimizing African spirituality, and reconstructing a society founded on African knowledge, power, and culture. Without transformative efforts, coloniality will continue to linger, shaping Nigerian life in unseen but pervasive ways. The journey toward decolonization is not just political; it is deeply epistemic, and every generation must contribute to breaking the invisible chains of coloniality.

## Data Availability

The raw data supporting the conclusions of this article will be made available by the authors, without undue reservation.
